# Decline in arylsulfatase B leads to increased invasiveness of melanoma cells

**DOI:** 10.18632/oncotarget.13751

**Published:** 2016-12-01

**Authors:** Sumit Bhattacharyya, Leo Feferman, Kaoru Terai, Arkadiusz Z. Dudek, Joanne K. Tobacman

**Affiliations:** ^1^ Department of Medicine, University of Illinois at Chicago, Chicago, IL 60612, USA; ^2^ Jesse Brown VA Medical Center, Chicago, IL 60612, USA

**Keywords:** arylsulfatase B, chondroitin 4-sulfate, galectin-3, invasiveness, malignant melanoma

## Abstract

Arylsulfatase B (ARSB; N-acetylgalactosamine 4-sulfatase) is reduced in several malignancies, but levels in melanoma have not been investigated previously. Experiments were performed in melanoma cell lines to determine ARSB activity and impact on melanoma invasiveness. ARSB activity was reduced ~50% in melanoma cells compared to normal melanocytes. Silencing ARSB significantly increased the mRNA expression of chondroitin sulfate proteoglycan(CSPG)4 and pro-matrix metalloproteinase(MMP)-2, known mediators of melanoma progression. Also, invasiveness and MMP activity increased when ARSB was reduced, and recombinant ARSB inhibited invasiveness and MMP activity. Since the only known function of ARSB is to remove 4-sulfate groups from the N-acetylgalactosamine 4-sulfate residue at the non-reducing end of chondroitin 4-sulfate (C4S) or dermatan sulfate, experiments were performed to determine the transcriptional mechanisms by which expression of CSPG4 and MMP2 increased. Promoter activation of CSPG4 was mediated by reduced binding of galectin-3 to C4S when ARSB activity declined. In contrast, increased pro-MMP2 expression was mediated by increased binding of the non-receptor tyrosine phosphatase SHP2 to C4S. Increased phospho-ERK1,2 resulted from SHP2 inhibition. Combined effects of increased C4S, CSPG4, and MMP2 increased the invasiveness of the melanoma cells, and therapy with recombinant ARSB may inhibit melanoma progression.

## INTRODUCTION

Malignant melanoma remains one of the most devastating malignancies, with propensity for widespread dissemination and resistance to treatment. Immunotherapy with checkpoint inhibition has produced some improvement in responses, but there is continuing need for new approaches to understanding how melanoma progresses and to develop effective interventions [1, 2]. The chondroitin sulfate proteoglycan 4 [CSPG4; also known as melanoma-associated chondroitin sulfate proteoglycan (MCSP) or neuron-glial antigen 2 (NG2)] was recognized as an important factor in the aggressiveness of melanoma decades ago, but successful intervention focused on CSPG4 has remained elusive [3–5]. In addition to the impact of CSPG4 on aggressiveness of melanoma, matrix metalloproteinase (MMP)-2, or gelatinase A, has also been associated with increased invasiveness of malignant melanoma, due to effects on the degradation of the extracellular matrix [6–8].

This report addresses the mechanism by which increased mRNA expression of CSPG4 and MMP2 occur following decline in the enzyme arylsulfatase B (ARSB; N-acetylgalactosamine 4-sulfatase). ARSB acts to remove 4-sulfate groups from the non-reducing end of chondroitin 4-sulfate (C4S) or dermatan sulfate (DS), and thereby initiates their degradation [9–11]. Previous work has shown that decline in ARSB and the resulting increase in chondroitin 4-sulfation were associated with transcriptional effects mediated by galectin-3 or by the ubiquitous non-receptor tyrosine phosphatase SHP2 [12–17]. Reduced binding of galectin-3 to the more highly sulfated C4S present when ARSB activity is less, leads to promoter activation due to interaction of galectin-3 with AP-1 (Activator protein-1) or Sp1 (Specificity protein 1). Increased expression of versican in human prostate epithelial and stromal cells, hypoxia inducible factor 1-alpha (HIF 1-α) in human colonic and bronchial epithelial cells, and Wnt9A (Wingless-Type MMTV Integration Site Family, Member 9A) in human colonic epithelial cells were reported.

In this report, decline in ARSB and the resultant increase in C4S contribute to increased expression of pro-MMP2 due to increased binding of SHP2 (PTPN11), a non-receptor tyrosine phosphatase, which binds more to the more highly 4-sulfated C4S present when ARSB is reduced [15]. Experiments presented in this report show how transcriptional events which follow decline in ARSB contribute to MMP activation and the invasive potential of melanoma cells through effects on SHP2 and phospho-ERK1,2.

Investigation of the role of the enzyme ARSB in other malignancies has shown that decline in ARSB is associated with more aggressive prostate and colon malignancies and that ARSB activity is reduced in malignant mammary cells and tissue compared to normal [18–22]. To assess how decline in ARSB might contribute to the aggressiveness of melanoma through effects on proteoglycan expression and signaling, the experiments presented in this report were performed in normal melanocytes and in several melanoma cell lines. The studies provide a basis for further assessment of the therapeutic impact of ARSB replacement on melanoma progression.

## RESULTS

### Progressive decrease in ARSB activity and increase in chondroitin 4-sulfate in more aggressive melanoma cell lines

**Figure 1:**
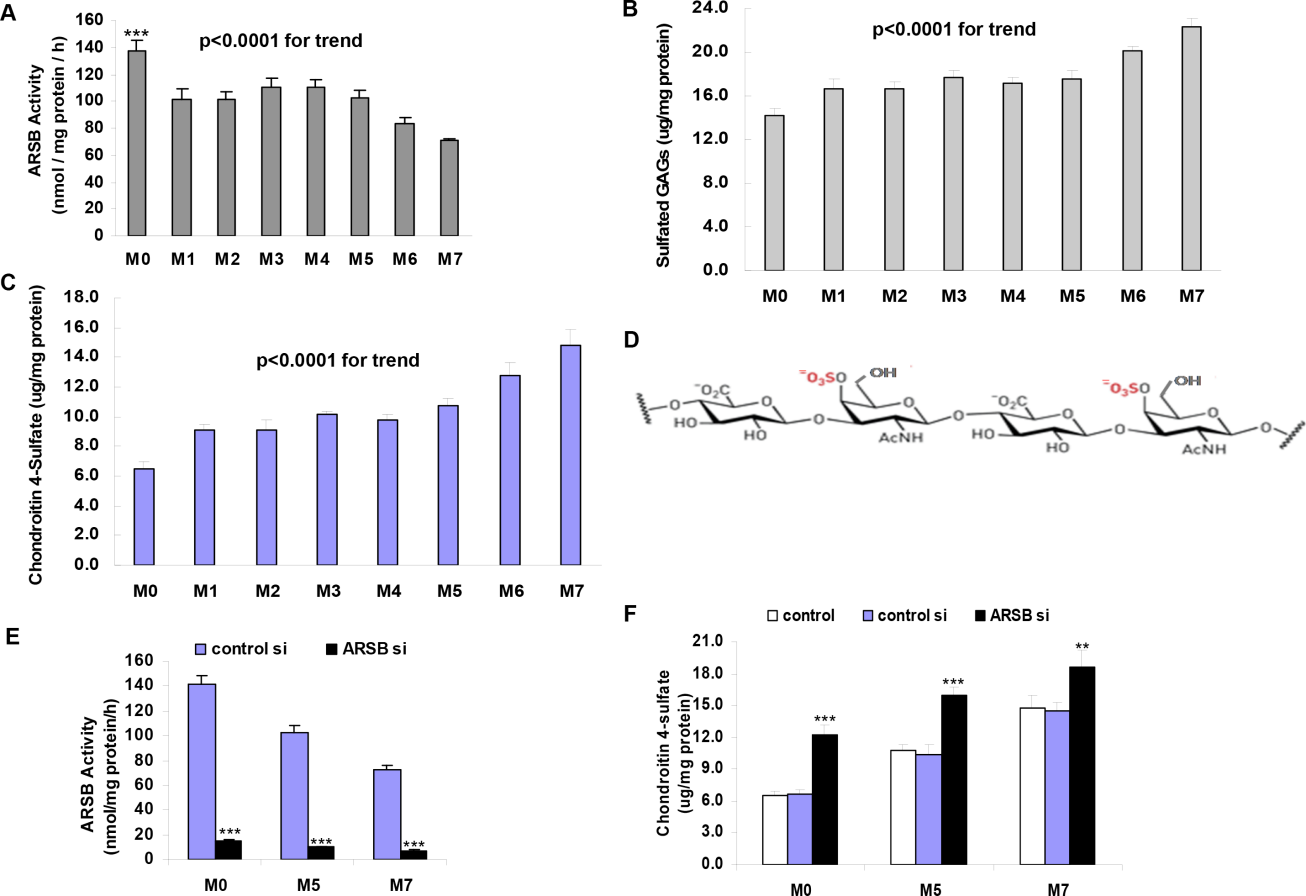
Decline in ARSB activity and increase in total sulfated glycosaminoglycans and C4S with increasing aggressiveness of melanoma cell lines (**A**) ARSB activity is significantly higher in the normal human melanocytes (M0; PCS-200-013, ATCC) than in the malignant cell lines ([WM1552C (M1), WM1552C/mock (mock CSPG4 transfection) (M2), WM1552C/MCSP (CSPG4 transfected) (M3), WM1552C/MCSPΔCD (transfected with CSPG4 without the cytoplasmic domain) (M4), WM35 (M5)], WM1341D (M6) and from metastatic melanoma (1205 Lu) (M7) (*p* < 0.001, one-way ANOVA with Tukey-Kramer pos*t*-test; *n* = 3). (**B**) Total sulfated glycosaminoglycans are greater in the more aggressive melanoma cell lines (*p* < 0.0001 for trend; *n* = 3). (**C**) Chondroitin 4-sulfate is significantly greater in the more aggressive melanoma cell lines, and the levels increase as the cell lines are increasingly aggressive (*p* < 0.0001 for trend; *n* = 3). (**D**) The structure of chondroitin 4-sulfate (C4S) shows: the presence of the 4-sulfate group of N-acetylgalactosamine; alternating beta-1, 3 and beta-1, 4 glycosidic bonds; and the fundamental disaccharide unit consisting of N-acetylgalactosamine 4-sulfate and glucuronate. (**E**) When ARSB is silenced by siRNA, the activity in the M0 cells declines to about 10% of the baseline level. Values in the M5 cells in the radial growth phase are intermediate between the values in the normal melanocytes (M0) and the values in the metastatic melanoma cell line (M7) (*n* = 3). (**F**) When ARSB is silenced by siRNA, the C4S level increases significantly in the three cell lines (*p* < 0.001; *n* = 3). [AcNH=N-acetyl; ARSB=arylsulfatase B; C4S=chondroitin 4-sulfate; GAG=glycosaminoglycan; si=siRNA=small interfering RNA].

Cell lines tested included normal melanocytes (designated M0), melanocytes from melanomas in radial growth phase (designated M1-M5), melanocytes from melanoma in vertical growth phase (M6), and melanocytes from metastatic melanoma (M7). ARSB activity declined from 138 ± 7 nmol/mg protein/h in the normal melanocytes to 71 ± 1 nmol/mg protein/h in the metastatic melanoma cell line (*p* < 0.001, 1-way ANOVA with Tukey-Kramer pos*t*-test for M0 vs. other cell lines) (Figure 1A). ARSB activity declined with increasing aggressiveness of the cell lines (*p* < 0.0001 for linear trend).

Consistent with the decline in ARSB activity, content of total sulfated glycosaminoglycans (GAGs) (Figure 1B) and of chondroitin 4-sulfate (C4S) (Figure 1C) increased with increased aggressiveness of the cell lines (*p* < 0.0001 for linear trend). Total sulfated GAGs increased from 14.1 ± 0.7 μg/mg protein in the M0 cells to 22.4 ± 0.8 μg/mg protein in the M7 cell line. The increase was largely attributable to the increase in C4S from 6.5 ± 0.4 μg/mg protein to 14.8 ± 1.1 μg/mg protein. The fundamental disaccharide unit of C4S, composed of N-acetylgalactosamine-4-sulfate residues linked to glucuronate residues, with alternating β-1,3 and β-1,4 glycosidic bonds, is shown (Figure 1D).

When ARSB was silenced by siRNA in the M0, M5, and M7 cell lines, ARSB activity declined to ~10% of the baseline level (Figure 1E). Consistent with the decline in ARSB, the C4S content in each of the cell lines increased significantly (*p* < 0.001) (Figure 1F).

### Chondroitin sulfate proteoglycan (CSPG)4 expression increased when ARSB activity declined

**Figure 2:**
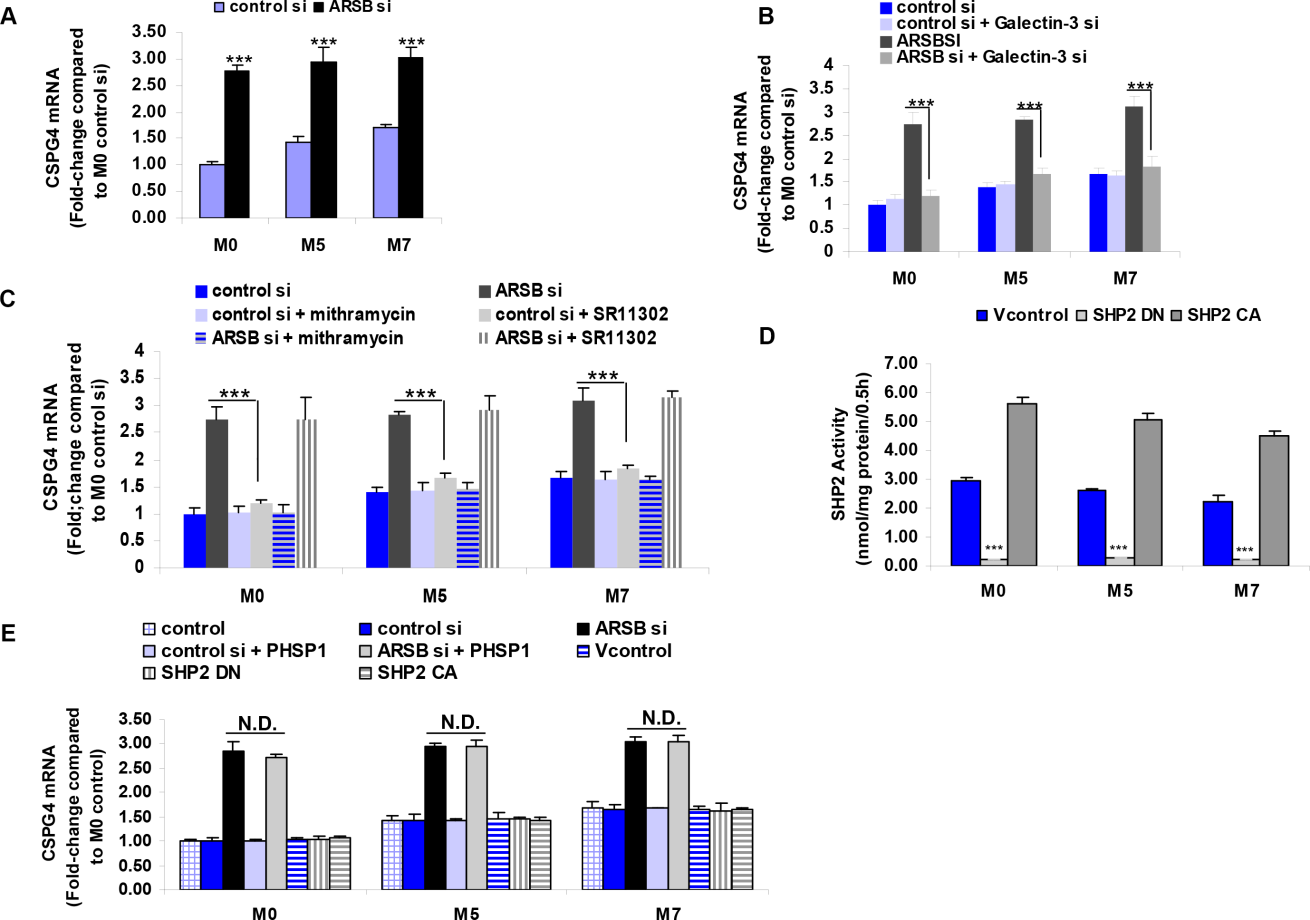
CSPG4 mRNA expression increases with decline in ARSB (**A**) CSPG4 expression is regulated by effects of the ARSB – C4S – galectin-3 – Sp1 pathway and is greater in the more aggressive melanoma cell lines (M5 and M7 vs. M0) (*p* < 0.001; *n* = 5). The increase in CSPG4 expression is associated with the decline in ARSB activity and the increase in C4S. (**B**) The increase in CSPG4 expression was inhibited when galectin-3 was silenced by siRNA (*p* < 0.001; *n* = 3). (**C**) Mithramycin, an inhibitor of Sp1 binding to DNA, inhibited the effect of ARSB siRNA on CSPG4 expression (*p* < 0.001; *n* = 3). In contrast, SR11302, an inhibitor of c-fos binding to DNA and of AP-1 mediated transcriptional effects, did not block the effect of ARSB siRNA on CSPG4 expression. (**D**) SHP2 activity was significantly increased by transfection with the constitutively active DNA construct and significantly reduced by the dominant negative construct (*p* < 0.001; *n* = 3). (**E**) SHP2 inhibition, by either PHSP1 or by dominant negative SHP2 DNA construct, had no effect on CSPG4 expression (*n* = 3). Also, the constitutive activation of SHP2 had no effect on CSPG4 expression. [AP-1=activator protein 1; ARSB=arylsulfatase B; CA=constitutively active; CSPG4=chondroitin sulfate proteoglycan 4; DN=dominant negative; N.D.=no difference; PHSP1=phenylhydrazonopyrazolone sulfonate; SHP2=tyrosine-protein phosphatase non-receptor type 11 (PTPN11); si=siRNA=small interfering RNA; Sp1=specificity protein 1; Vcontrol=vector control].

CSPG4 expression was determined by QRT-PCR in the M0, M5, and M7 cell lines. Expression in the metastatic M7 cell line was ~1.7 times the level in the M0 cells at baseline (Figure 2A). When ARSB was silenced by siRNA, the CSPG4 expression increased to about three times the baseline level in each of the cell lines (*p* < 0.001) (Figure 2A).

Since previous investigation indicated that transcriptional events initiated by decline in ARSB were mediated by reduced binding of galectin-3 to the more highly sulfated C4S that was present when ARSB activity was less, the effect of galectin-3 silencing on CSPG4 expression was assessed. When galectin-3 was silenced, the increase in CSPG4 expression was completely inhibited (*p* < 0.001) (Figure 2B).

The impact of galectin-3 on transcription was previously shown to be mediated by interaction with transcription factors AP-1 or Sp1 [12–14]. To address whether AP-1 or Sp1 was involved in the ARSB-C4S-galectin-3 induced increase in CSPG4 expression, the impact of the AP-1 inhibitor SR11302 and of the Sp1 inhibitor mithramycin was tested. SR11302 had no effect, but mithramycin exposure completely inhibited the ARSB silencing induced increase in CSPG4 (*p* < 0.001) (Figure 2C).

The tyrosine phosphatase SHP2 also can mediate effects of ARSB and C4S on transcription, due to increased binding of SHP2 with more highly sulfated C4S when ARSB is reduced. The SHP2 DN and CA DNA constructs significantly modified the SHP2 activity in the M0, M5, and M7 cell lines and in the anticipated direction (Figure 2D). By trypan blue exclusion, the SHP2 DN and CA constructs had no impact on the viability of the melanoma cells at 24 h. The effects of SHP2 dominant negative (DN) and constitutively active (CA) DNA constructs and of the SHP2 inhibitor PHSP1 were tested and shown to have no effect on CSPG4 expression (Figure 2E).

### CSPG4 promoter activation following ARSB and galectin-3 silencing

**Figure 3:**
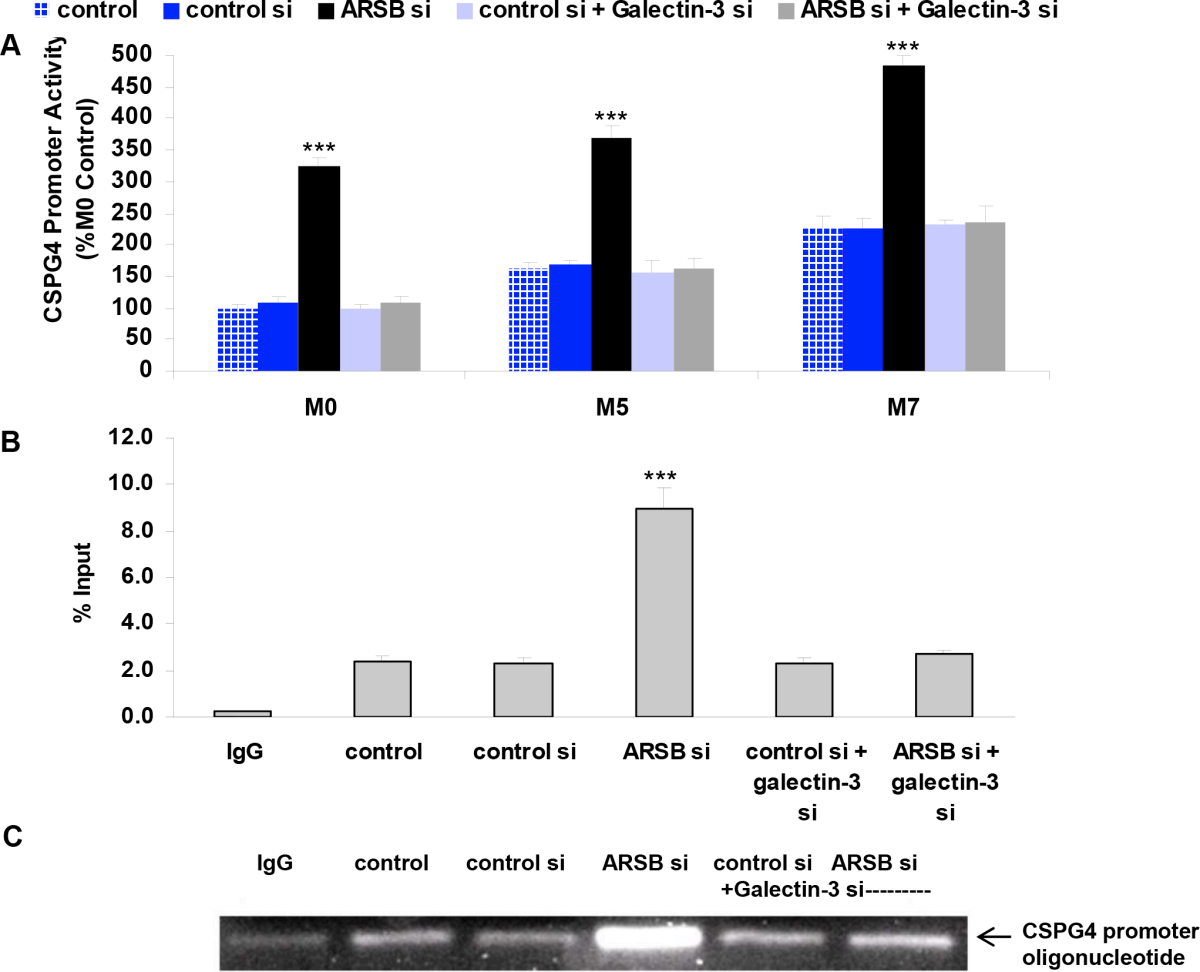
CSPG4 promoter activation increased following ARSB knockdown (**A**) CSPG4 promoter activation was least in the normal melanocytes (M0), compared to the radial growth phase melanoma (M5) or metastatic melanoma (M7) cells. ARSB silencing increased the luciferase activity, and galectin-3 silencing inhibited the promoter activation (*p* < 0.001, *n* = 5). (**B**) Chromatin immunoprecipitation (ChIP) using an Sp1 antibody and following ARSB silencing showed increased binding to the CSPG4 promoter region, which encompassed an Sp1 binding site. Galectin-3 silencing inhibited the ARSB-silencing induced increase (*p* < 0.001; *n* = 3). (**C**) Blot shows increased density of the Sp1 binding to the CSPG4 promoter following ARSB silencing. The blot shows reduced density following galectin-3 silencing. [ARSB=arylsulfatase B; ChIP=chromatin immunoprecipitation; CSPG4=chondroitin sulfate proteoglycan 4; si=siRNA=small interfering RNA; Sp1=Specificity protein 1].

The activation of the CSPG4 promoter following ARSB- and galectin-3 silencing was determined using a luciferase-tagged promoter construct. At baseline, promoter activation was significantly higher in the M5 cells than in the normal melanocytes, and higher in the metastatic M7 cells than in the M5 cells (*p* < 0.01, *p* < 0.01) (Figure 3A). Marked activation of the promoter followed ARSB silencing in the M0, M5, and M7 cells, but declined when galectin-3 was silenced, consistent with a requirement for galectin-3 for the transcriptional effect (Figure 3A).

Chromatin immunoprecipitation assay using the M0 cells and Sp1 antibody confirmed the binding of Sp1 to the CSPG4 promoter (Figure 3B), and showed that the effects of ARSB silencing were nullified when galectin-3 was silenced. Immunoblot demonstrated increased intensity of binding of Sp1 to its consensus sequence in the CSPG4 promoter following ARSB silencing, and inhibition of this increase following galectin-3 silencing (Figure 3C).

### Pro-MMP2 expression increased following ARSB silencing due to SHP2-mediated effects

**Figure 4:**
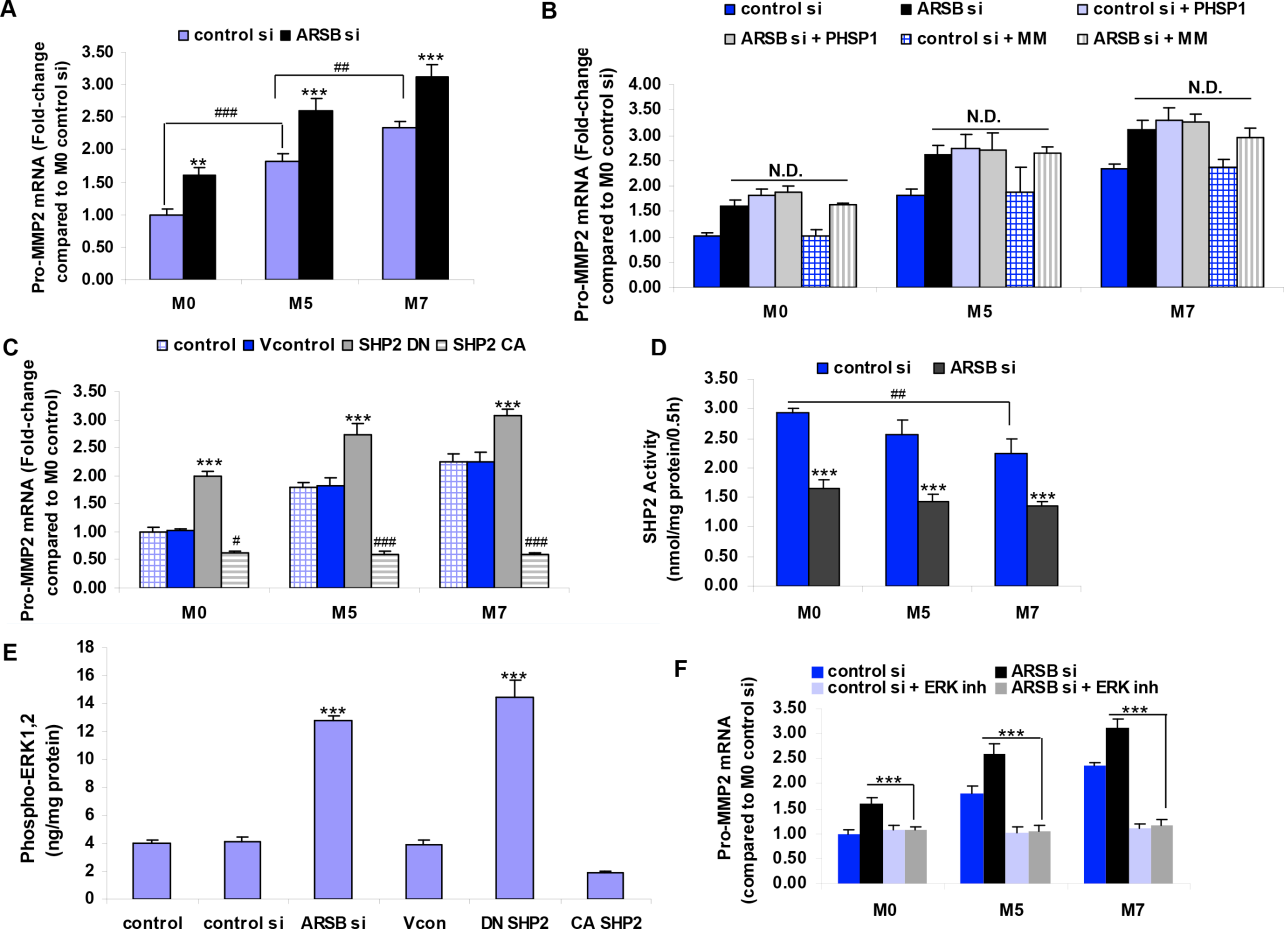
Increase in pro-MMP2 mRNA expression following ARSB silencing is mediated by SHP2 and by phospho-ERK1,2 (**A**) Pro-MMP2 expression increased when ARSB was silenced in the M0, M5, and M7 cells (*** for *p* < 0.001, ** for *p* < 0.01; *n* = 5). Baseline pro-MMP2 expression was significantly greater in the M5 and M7 cells than in the M0 cells (### for *p* < 0.001, ## for *p* < 0.01; *n* = 5). (**B**) Exposure to mithramycin (MM) did not inhibit the ARSB-induced increased in pro-MMP2 expression. The SHP2 inhibitor PHSP1 also increased the pro-MMP2 expression, and the combination of ARSB silencing and PHSP1 did not further increase the pro-MMP2 mRNA expression. (**C**) Pro-MMP2 expression increased following transfection by the dominant negative SHP2 DNA construct and declined from baseline following transfection by the constitutively active SHP2 construct (*** for *p* < 0.001, ### for *p* < 0.001, # for *p* < 0.05; *n* = 5). (**D**) ARSB silencing reduced the SHP2 activity in the M0, M5, and M7 cell lines (*** for *p* < 0.001; *n* = 3). The M0 control si level of SHP2 activity was significantly higher than in the control si M7 cells (## for *p* < 0.01; *n* = 3). (**E**) Phospho-ERK1,2 was markedly increased following ARSB silencing and transfection by the DN SHP2 construct in the M0 cells (*p* < 0.001; *n* = 3). (**F**) Pro-MMP2 expression was significantly reduced following exposure to an ERK inhibitor (ERK activation inhibitor peptide 1) (*p* < 0.001; *n* = 5). [ARSB=arylsulfatase B; CA=constitutively active; CSPG4=chondroitin sulfate proteoglycan 4; DN=dominant negative; ERK=extracellular signal-regulated kinase; MMP=matrix metalloproteinase; inh=inhibitor; MM=mithramycin; N.D.=no difference; PHSP1= phenylhydrazonopyrazolone sulfonate; SHP2= tyrosine-protein phosphatase non-receptor type 11 (PTPN11); si=siRNA=small interfering RNA; Vcon=vector control].

Pro-MMP2 mRNA expression was significantly greater in the M5 and M7 cells than in the M0 cells (*p* < 0.001). When ARSB was silenced in the M0, M5, and M7 cells, the expression of pro-MMP2 increased significantly (*p* < 0.001), compared to control silencing (Figure 4A). At baseline, the mRNA expression of MMP9 was significantly greater in the M5 cells than in the M0 cells (*p* < 0.001) and in the M7 cells than in the M5 cells (*p* < 0.05). Following ARSB silencing, the MMP9 expression increased significantly in each of the cell lines, rising to 2.94 ± 0.09 times the baseline level in the M0 cells. However, the cycle threshold (C_t_) for MMP9 was ~30.4 in the control si M0 cells, considerably higher than the value for MMP2 (C_t_ = ~20.4) and indicative of ~1000-fold less mRNA expression of MMP9 than of MMP2.

Further assessment of the mechanism for increased expression of pro-MMP2 showed that mithramycin, an inhibitor of Sp1-mediated transcription, did not affect the pro-MMP2 expression. However, treatment with the SHP2 inhibitor PHSP1 increased pro-MMP2 expression (Figure 4B). The dominant negative (DN) SHP2 DNA construct also increased the MMP2 expression, whereas the constitutively active (CA) SHP2 construct inhibited MMP2 expression (Figure 4C). SHP2 activity was significantly inhibited by ARSB silencing in the melanocytes, and SHP2 activity was greatest in the normal melanocytes which had the highest ARSB activity (Figure 4D). These experiments indicated that decline in ARSB increased the pro-MMP2 expression through inhibition of SHP2.

ARSB knockdown and dominant negative SHP2 increased the phospho-ERK1,2 in the normal melanocytes (Figure 4E). Pro-MMP-2 expression declined in the M0, M5, and M7 cells following treatment with the ERK activation inhibitor peptide 1 (Figure 4F). These results are consistent with a requirement for phospho-ERK1,2 in the ARSB siRNA- and SHP2 DN- mediated increase in pro-MMP2 expression.

### MMP2 activity and invasiveness were increased by decline in ARSB and reduced by recombinant ARSB

**Figure 5:**
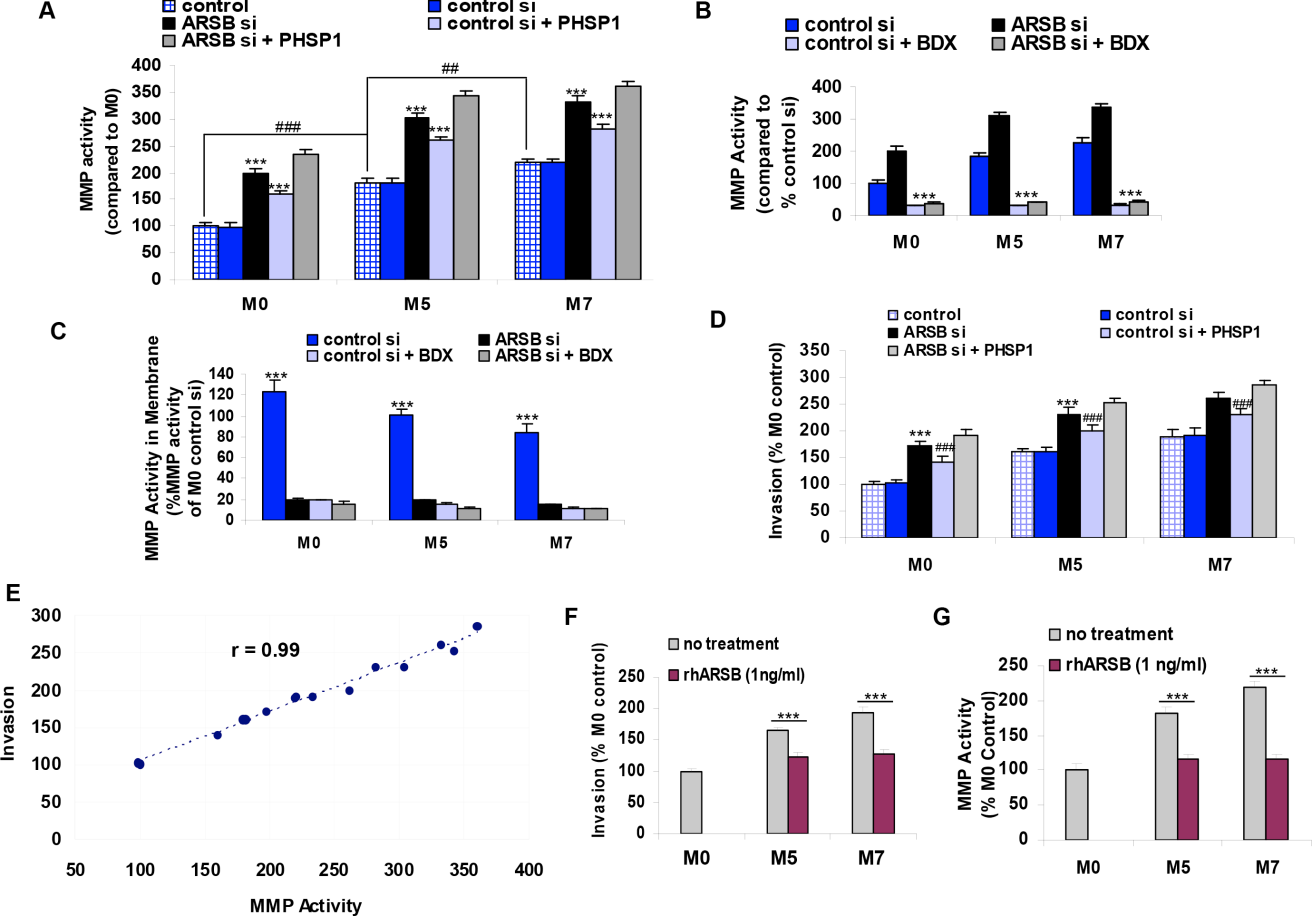
Matrix metalloproteinase activity and invasiveness of melanoma cells are increased by decline in ARSB and reduced by recombinant ARSB. (A) MMP activity increased when ARSB was silenced and when cells were treated with the SHP2 inhibitor PHSP1 (*** for *p* < 0.001; *n* = 3). MMP activity was significantly higher in the control M5 cells than in the M0 control (### for *p* < 0.001), and in the control M7 cells than in the control M5 cells (## for *p* < 0.01). The combination of ARSB siRNA and PHSP1 led to further increases in MMP activity compared to either treatment alone (*p* < 0.01 for M0, *p* < 0.001 for M5, and *p* < 0.05 for M7; *n* = 3). (**B)** Decline in ARSB increased the MMP activity in the spent media in the M0, M5, and M7 cells, as in A. The increases following ARSB silencing were blocked by treatment with β-D-xyloside, an inhibitor of chondroitin sulfate proteoglycan biosynthesis (*p* < 0.001; *n* = 3). The baseline level of MMP activity was also inhibited by exposure to β-D-xyloside. (**C**) Membrane-associated MMP activity was measured following exposure to β-D-xyloside, ARSB silencing, and their combination. In association with the increase in MMP activity in the spent media when ARSB was silenced, the membrane MMP activity following control silencing was significantly greater than the membrane MMP activity following ARSB knockdown (*p* < 0.001). Exposure to the β-D-xyloside inhibited the MMP activity in both control and ARSB-silenced cell membranes (*p* < 0.001; *n* = 3). (**D)** Invasiveness of the melanocytes was detected by fluorescence of cells which migrated to the underside of an ECM-coated filter. Invasiveness was significantly increased by ARSB silencing and by exposure to the SHP2 inhibitor PHSP1 (*p* < 0.001; *n* = 5). **(E)** The correlation r between MMP activity and invasiveness was 0.99. (**F)** Recombinant human ARSB inhibited the invasiveness of the M5 melanoma cells by >40% and of the M7 metastatic melanoma cells by >65% (*p* < 0.001; *n* = 5). **(G)** MMP activity declined ~67% in the M5 cells and ~100% in the M7 cells following exposure to recombinant human ARSB (*p* < 0.001; *n* = 5). [ARSB=arylsulfatase B; BDX=β-D-xyloside=methyl β-D-xylopyranoside; ECM=extracellular matrix; MMP = matrix metalloproteinase; PHSP1=phenylhydrazonopyrazolone sulfonate; rhARSB=recombinant human ARSB; si=siRNA=small interfering RNA].

MMP2 activity measurements were performed under conditions that favored the activation of MMP2. The MMP2 activity in the spent media was greatest in the M7 cell line, compared to the M0 and M5 cells (*p* < 0.001) (Figure 5A). Both ARSB knockdown and SHP2 inhibition by PHSP1 increased the MMP2 activity. The combination of ARSB siRNA and PHSP1 further increased the MMP2 activity (Figure 5A).

MMP2 activity in the spent media increased following ARSB silencing and declined markedly following exposure to β-D-xyloside, an inhibitor of chondroitin sulfate proteoglycan biosynthesis (Figure 5B). The MMP2 activity in the cell membranes declined markedly following either ARSB silencing or treatment with β-D-xyloside (Figure 5C) (*p* < 0.001, *n* = 3). The marked decline in the MMP2 activity following β-D-xyloside is consistent with a requirement for the presence of CSPG4/C4S as a platform for the activation of MMP2.

Invasiveness of the melanoma cells increased following ARSB silencing or treatment with the SHP2 inhibitor PHSP1 (*p* < 0.001). Invasion was further increased by the combination of ARSB silencing and PHSP1 (Figure 5D). The correlation coefficient r between MMP activity and invasiveness was 0.99 (Figure 5E). Both invasiveness (Figure 5F) and MMP activity (Figure 5G) were significantly reduced by exogenous recombinant human ARSB.

**Figure 6:**
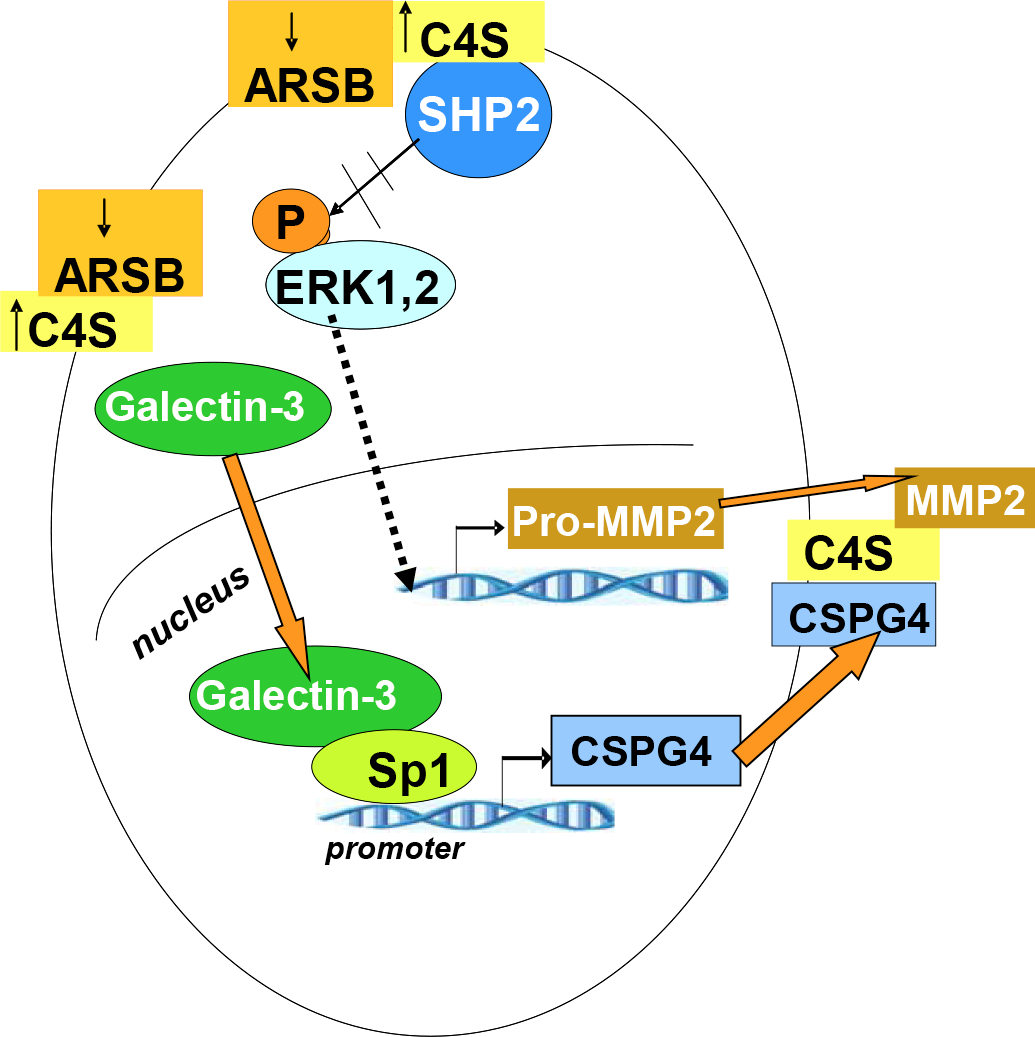
Overall schematic of pathway by which decline in ARSB leads to increases in CSPG4 and MMP2 Two pathways by which decline in ARSB lead to increased expression of mediators of melanoma invasiveness are shown. Expression of CSPG4 is increased due to reduced binding of galectin-3 to the more highly sulfated C4S present when ARSB activity is less. This leads to activation of a galectin-3 and Sp1-mediated increase in the expression of CSPG4, a cell surface proteoglycan with C4S attachments. In addition to increased CSPG4, decline in ARSB and the associated increase in C4S, lead to increased binding of the non-receptor tyrosine phosphatase SHP2 to the more highly sulfated C4S. Decline in SHP2 activity follows, and leads to prolonged activation of phospho-ERK1,2 and subsequent enhancement of pro-MMP2 expression. Pro-MMP2 attaches to C4S chains on CSPG4 and is activated by MT1-MMP (MMP14). MMP2 can then act as a gelatinase/collagenase and modify the extracellular matrix, facilitating the migration and invasion of the melanoma cells. [ARSB=arylsulfatase B; C4S=chondroitin 4-sulfate; CSPG4=chondroitin sulfate proteoglycan 4; ERK=extracellular signal-regulated kinase; MMP = matrix metalloproteinase; MT1-MM = membrane type 1 metalloproteinase; Sp1=specificity protein 1].

The effects of decline in ARSB on melanoma invasiveness are presented schematically in Figure 6. This schematic shows two major effects. First, when ARSB is silenced and the chondroitin 4-sulfation thereby increased, galectin-3 binds less to C4S and enhances the mRNA expression of CSPG4 through a transcriptional mechanism involving Sp1. Second, when ARSB is silenced and chondroitin 4-sulfation increased, the tyrosine phosphatase SHP2 binds more to C4S, leading to sustained ERK1,2 phosphorylation and subsequent transcriptional events, including increased expression of pro-MMP2. Pro-MMP2 migrates to C4S chains which are attached to CSPG4. Pro-MMP2 undergoes activation, and contributes to invasiveness by remodeling of the ECM.

## DISCUSSION

In this report, we present new mechanistic insights into how melanoma progression can arise in relation to decline in the enzyme arylsulfatase B, with concomitant increase in chondroitin 4-sulfation. Chondroitin 4-sulfate (C4S) is very abundant in human tissues, present in ug/mg quantities, so transcriptional effects arising due to more or less binding with the 4-sulfate group of the non-reducing end of C4S can have profound effects on vital cell processes. The studies reported here show marked increase in expression of CSPG4 and of pro-MMP2 following decline in ARSB.

Both CSPG4 and MMP2 have previously been implicated in melanoma progression [3–8]. CSPG4 was associated with melanoma aggressiveness decades ago, and therapeutic efforts directed at inhibition of CSPG4 have been undertaken. CSPG4, also known as melanoma- associated chondroitin sulfate proteoglycan (MCSP), has been extensively described in the melanoma literature and its expression associated with disease progression [23–26]. Since CSPG4 has C4S attachments and CSPG4 expression increased when ARSB activity was less, the current studies suggest a mechanism whereby C4S localization may be regulated by changes in C4S sulfation. CSPG4 is not the only proteoglycan with C4S attachments, and other chondroitin sulfate proteoglycans, such as versican, may also be increased in the melanoma cells [27, 28]. The increase in C4S due to decline in ARSB leads to increase in CSPG4 expression through the galectin-3/Sp1 transcriptional mechanism. When CSPG4 is overexpressed by transfection of a CSPG4 construct in the WM1552C/MCSP (CSPG4 transfected) M3 cells [29, 30], and the overexpression is independent of promoter activation, the C4S content was not significantly increased, in comparison to the values in the WM1552C/mock (mock CSPG4 transfection) (M2) cells or the WM1552C/MCSPΔCD (transfected with CSPG4 without the cytoplasmic domain) M4 cells (Figure 1B).

The increased expression of CSPG4, as well as the impaired degradation of C4S when ARSB is deficient, can provide an expanded platform for pro-MMP2 activation and remodeling of the ECM [31]. MMP2 has been identified in multiple malignancies and associated with poor prognosis [32, 33]. Membrane Type 1 Matrix Metalloproteinase (MT1-MMP; MMP14) activates pro-MMP2, which is attached to C4S chains of CSPG4. In addition to the observed effects on mRNA expression of CSPG4 and pro-MMP2, the expression of another gelatinase, MMP9, was also increased in the malignant melanoma cells and following silencing of ARSB. Multiple other MMPs have also been reported to be increased in melanoma cells and tissue, including MMPs 1, 8, 13, 14, 15, 16, 17, and 19 [34–36].

Experiments in this report have shown that ARSB silencing and decline in SHP2 increase phospho-ERK1,2 in the melanocytes and that ERK inhibition reduces the ARSB siRNA-induced increase in pro-MMP2 expression. At baseline, there was a significant increase in the pro-MMP2 expression in the malignant cells, compared to the normal melanocytes. ARSB silencing or SHP2 DN further increased the pro-MMP2 expression. However, the greater decrease in SHP2 activity by the SHP2 DN construct, compared to by ARSB knockdown, did not lead to greater increase in pro-MMP2 expression in the malignant cells (Supplementary Table S1). Differences persisted between the M0, M5, and M7 cells in the pro-MMP2 expression, suggesting that other endogenous, pre-existing molecular modifications, perhaps initiated by the pre-existing decline in SHP2 activity in the M5 and M7 cells, contribute to the increase in pro-MMP2 expression. The combined effects of decline in ARSB on the increased expression of CSPG4 proteoglycan and of pro-MMP2 contribute to the increased invasiveness of melanoma cells [37]. The inhibition of the non-receptor tyrosine phosphatase SHP2 can lead to profound effects on phosphorylations following ARSB decline. Targeted intervention at the level of ARSB can modify a broad spectrum of signaling pathways, and may help to achieve control of malignant progression and restoration of normal cell-matrix interaction. Future experiments will help to clarify the underlying mechanisms involving sulfatases, phosphatases, proteases, and chondroitin sulfate proteoglycans, and to identify how the interactions among these different molecules contribute to melanoma progression.

## MATERIALS AND METHODS

### Cell culture of melanocytes and melanoma cell lines

Normal melanocytes (ATCC PCS 200–013; referred to as M0) and melanoma cell lines in radial or vertical growth phase were cultured under the recommended conditions [29, 30]. Cell lines included: WM1552C (M1), WM1552C/mock (mock CSPG4 transfection) (M2), WM1552C/MCSP (CSPG4 transfected) (M3), WM1552C/MCSPΔCD (transfected with CSPG4 without the cytoplasmic domain) (M4), WM35 (M5), WM1341D (M6) and from metastatic melanoma (1205 Lu) (M7). M1-M5 and M7 cell lines had the V600E BRAF mutation; the M6 cell line had the V600R BRAF mutation. Primary normal melanocytes (M0) were cultured in dermal cell basal medium (ATCC, Manassas, VA) with adult melanocyte growth kit (ATCC). Media for malignant cells was MCDB153 (80%; Sigma Chemical Co, St. Louis, MO), L-15 (20%; Sigma) with 2% FBS, Insulin (5 μg/ml; Sigma) and CaCl_2_ (1.68 mM). Cells were grown in T75 or T25 flasks or in 24-well cell culture clusters with media replenishment every 48 hours. Cells were harvested by scraping.

### QRT-PCR of CSPG4, MMP2, and MMP9

QPCR of CSPG4 and MMP2 was performed using standard procedures and the following primers: for CSPG4 (NM_001897) Left: cttcaactacagggcacaagg and Right: aggacattggtgaggacagg; for MMP2 (NM_004530) left: AG TGGATGATGCCTTTGCTC and right: GAGTCCGTCCT TACCGTCAA; and for MMP9 (NM_004994.2) left: GTCT TCCCCTTCACTTTCCTG and right: TCAGTGAAGCG GTACATAGGG. Β-actin expression was used as control, as previously [13].

### ARSB activity assay and measurement of total sulfated glycosaminoglycans and chondroitin 4-sulfate

ARSB activity assay was performed with the exogenous substrate 4-methylumbelliferylsulfate, using a fluorometric assay and following a standard protocol, as previously reported [20]. Measurements of total sulfated glycosaminoglycans (GAGs) and chondroitin 4-sulfate (C4S) were performed using 1,9-dimethylmethylene blue dye (Blyscan^TM^ Biocolor Ltd, Newtownabbey, N. Ireland), as previously [13].

### Silencing of ARSB and galectin-3 by siRNA

Small interfering RNA was obtained to knockdown the expression of ARSB and galectin-3 (Qiagen, Germantown, MD). Effects on mRNA were determined by QRT-PCR. The siRNA sequences for ARSB (NM_000046) silencing were: sense 5′- GGGUAUGGUCUCUAGGCA - 3′ and antisense: 5′- UUGCCUAGAGACCAUACCC - 3′. The sequence of the DNA template for human galectin-3 silencing (Hs_LGALS3_9) was: 5′ - ATGATGTTGCCTTCCACTTTA - 3′. Cells were grown to ~60% confluence, then silenced by adding 0.6 μl of 20 μM siRNA (150 ng), mixed with 100 μl of serum-free medium and 12 μl of HiPerfect Transfection Reagent (Qiagen), as previously [14]. Effectiveness of ARSB silencing was confirmed by activity assay, with decline of ~90%. Galectin-3 silencing was confirmed by galectin-3 ELISA, with galectin-3 protein declining ~ 90%.

### Treatment with SHP2 DNA constructs and recombinant ARSB

SHP2 dominant negative (DN), constitutively active (CA), wild type (WT), and empty vector plasmids were obtained (from Dr. Stuart Frank, University of Alabama at Birmingham) [38] and transfected into the melanoma cells by Lipofectamine™ 2000 (Invitrogen, Carlsbad, CA). Efficiency of transfection at 24 h was determined by measurements of SHP2 protein or SHP2 activity by ELISA (R&D, Minneapolis, MN). Cell viability at 24 h following treatment with the SHP2 DN and CA constructs was determined by trypan blue exclusion [39]. Recombinant human ARSB was obtained (R&D), and cultured cells were treated with 1 ng/ml x 24 h.

### Matrix metalloproteinase (MMP) activity

Matrix metalloproteinase (MMP) activity was measured in the spent media using the MMP activity assay (Abcam, Cambridge, MA) in which a fluorescence resonance energy transfer (FRET) peptide is the MMP substrate. In the intact FRET peptide, the endogenous fluorescence of one part of the peptide is quenched by another part of the peptide. Following cleavage into two fragments by MMP, the fluorescence is recovered and detected in the fluorescence microplate reader (FLUOstar, BMG Labtech, Cary, NC) at Ex/Em = 490/525 nm. Melanocytes were grown in 24-well clusters, and the FRET peptide was added when cells were ~70% confluent. Conditions favored the detection of MMP2 activity, incubating the samples with an equal volume of 1 mM APMA (4-aminophenylmercuric acetate) at 37°C for 1 h.

### Matrix metalloproteinase (MMP) activity in cell membranes

Cell membranes were prepared following a standardized protocol [40]. The MMP activity in the samples was activated by incubation with an equal volume of 1 mM APMA (4-aminophenylmercuric acetate) for 1 h at 37°C. Following activation, samples were kept at room temperature for 10–15 minutes, MMP green substrate solution was added, and samples were incubated at room temperature in the dark for 1 h. MMP activity was detected by fluorescence at Ex/Em 490/525 nm and compared to determinations of MMP activity from the spent media of the M0 cell preparation exposed to control siRNA.

### Invasiveness of melanocytes

Melanocyte invasiveness was detected using a fluorescent cell invasion assay (QCM^TM^ ECMatrix cell invasion assay, Chemicon, EMD Millipore). A 96-well cell culture plate and cell culture inserts with 8 μm pores in a polycarbonate membrane and coated with a thin, dried layer of ECMatrix^TM^ to block non-invasive cell migration, were used. The invasive melanoma cells migrated through the ECM layer and attached to the bottom of the polycarbonate membrane. The cells were dissociated from the membrane by incubation with Cell Detachment Buffer. Then, the invaded cells were lysed and detected by a green-fluorescent dye (CyQuant GR dye, Molecular Probes) using a fluorescence plate reader (BMG).

### CSPG4 promoter activation

The CSPG4 promoter sequence with an Sp1 binding site (GCCCCGCCCC) was identified using online resources [alggen-promo. http://alggen.lsi.upc.es/cgi-bin/promo_v3/promo/promoinit.cgi?dirDB=TF_8.3]. The promoter construct was tagged with a *Renilla renformis* luciferase reporter gene (RenSP) to detect activation of the promoter following silencing of ARSB, or other treatment in the melanocytes (LightSwitch Assay, SwitchGear Genomics, Menlo Park, CA). The β-actin promoter (GoClone^TM^) construct with RenSP was the positive control, and a scrambled sequence (R01) with RenSP was the negative control; these were used to determine the effectiveness and specificity of the transfections. Transfections were performed with cells at 70% confluence, following silencing for 24 h, with FuGENE HD transfection reagent and proprietary LightSwitch Assay Reagent (SwitchGear). Luminescence was read at 480 nm in a microplate reader (BMG) after incubation for 24 hours, and compared among the different cell preparations.

### Treatment with inhibitors of galectin-3, AP-1, Sp1, SHP2, ERK1,2, and chondroitin sulfate proteoglycan biosynthesis

Galectin-3, AP-1, Sp1, and SHP2 mediated the transcriptional effects of ARSB due to reduced binding of galectin-3 to more highly sulfated C4S present when ARSB was reduced or to enhanced binding of SHP2 with C4S when ARSB activity was reduced. Melanocytes were treated with specific inhibitors of AP-1, Sp1, and SHP2, or with galectin-3 siRNA, which were previously shown to affect promoter activation following ARSB silencing, and the effect on CSPG4 expression was determined. After silencing by siRNA, cells were treated with SR11302 (5 μM × 24 h, Santa Cruz Biotechnology), a retinoid that inhibits AP-1 transcription factor activity by blocking binding of c-fos to the AP-1 consensus sequence [14], mithramycin (250 nM × 24 h); Sigma), an inhibitor of Sp1 binding to DNA [13]; and PHSP1 (phenylhydrazonopyrazolone sulfonate; 30 μM × 24 h; Sigma), a chemical inhibitor of SHP2 [15]. Effects of SHP2 dominant negative (DN) and constitutively active (CA) DNA constructs [38] were also tested. ERK activation inhibitor peptide 1 (10 μM × 24 h; Sigma) was used to inhibit the activation of ERK1,2. Effects of methyl β-D-xylopyranoside (1 mM × 18 h after ARSB silencing by siRNA x 6 h; Sigma), an inhibitor of glycosaminoglycan elongation on the core protein of proteoglycans, on the mRNA expression of MMP2 and MMP activity were determined by QRT-PCR and MMP activity assay. Pro-MMP in the cell membranes was activated by APMA (4-aminophenylmercuric acetate; 1 mM; Abcam) for 1 h at 37°C. Then, MMP activity was measured and compared to the MMP activity in the media of the cell preparations following exposure to ARSB siRNA and/or β-D-xyloside.

### Chromatin immunoprecipitation assay to detect Sp1 binding to the CSPG4 promoter

Sp1 and galectin-3 binding to the CSPG4 promoter were assessed by chromatin immunoprecipitation (ChIP) assay. The CSPG4 promoter sequence was accessed (SwitchGear), and a putative Sp1 binding site was identified in the CSPG4 promoter [alggen-promo. http://alggen.lsi.upc.es/cgi-bin/promo_v3/promo/promoinit.cgi?dirDB=TF_8.3]. Primers which encompassed the Sp1 binding site (GCCCCGCCCC) were selected [Primer 3 http://bioinfo.ut.ee/primer3/ 4.0.0]. Primers were: (left) GGGCCCTTTAAGAAGGTTGA and (right) AGCTGGGAGCTGAGTGGAG). For ChIP assay, normal melanocytes were treated with IgG as control, control siRNA, ARSB siRNA, or the Sp1 inhibitor mithramycin. Melanocytes were fixed with 1% formaldehyde for 10 min at room temperature, followed by sonication to shear the chromatin (ChIP Assay, Active Motif, Carlsbad, CA). The sheared DNA was incubated with rabbit polyclonal anti-galectin-3 (sc-20157, SCBT, Santa Cruz, CA) and anti-Sp-1 (Active Motif) antibodies for 1 h, as well as with IgG control. Protein–DNA complexes were precipitated by protein A/G-coupled magnetic beads, and the DNA was purified from the immunoprecipitated complexes by reversal of cross-linking, followed by proteinase K treatment. Then, real-time RT-PCR was performed using Brilliant SYBR Green QRT-PCR master mix (Stratagene, La Jolla, CA) and Mx3000 (Stratagene) to amplify the CSPG4 promoter. Band intensity was compared among the ARSB silenced, galectin-3 silenced, and combined ARSB- silenced and galectin-3-silenced preparations, as well as IgG, untreated control, and control siRNA preparations on a 1.5% agarose gel.

### Statistics

All experiments were performed with at least three independent biological samples and with technical replicates of each measurement. Control assays were performed together with the test assays. Statistical analysis was performed using one-way ANOVA with Tukey-Kramer pos*t*-test (InStat, GraphPad Software, San Diego, CA), unless stated otherwise. Analysis for trend was performed using InStat software. Significance was determined by *p*-value with *p* < 0.05 significant and shown by *p < 0.01 was designated by ** and *p* < 0.001 by ***. Pearson's correlation coefficient r was determined using Excel software.

## SUPPLEMENTARY TABLE


