# Supplementary material for: Decline in arylsulfatase B leads to increased invasiveness of melanoma cells
Source: Oncotarget. 2016 Dec 1;8(3):4169–80. doi: 10.18632/oncotarget.13751 (PMC5354821; doi:10.18632/oncotarget.13751)
Supplement: Supplementary file 1 [file oncotarget-08-4169-s001.pdf]

Decline in arylsulfatase B leads to increased invasiveness of melanoma cells

Supplementary Materials

Supplementary Table S1: Effects of ARSB silencing and SHP2 dominant negative construct on SHP2 activity and on MMP2 mRNA expression

Treatment	M0	M5	M7
	**SHP2 Activity (nmol/mg protein/0.5h) (S.D.)**		
ARSB si	1.65 (0.16)	1.44 (0.12)	1.36 (0.07)
Control si	2.94 (0.08)	2.55 (0.24)	2.25 (0.24)
SHP2 DN	0.22 (0.02)	0.26 (0.02)	0.22 (0.02)
Vector control	2.93 (0.14)	2.62 (0.06)	2.25 (0.18)
	**Pro-MMP2 mRNA (fold-change compared to M0 control) (S.D.)**		
ARSB si	1.61 (0.11)	2.60 (0.19)	3.12 (0.19)
Control si	1.00 (0.08)	1.82 (0.13)	2.35 (0.08)
SHP2 DN	2.00 (0.08)	2.73 (0.20)	3.08 (0.11)
Vector control	1.01 (0.04)	1.82 (0.13)	2.23 (0.17)
